# PRC2-dependent regulation of ganglioside expression during dedifferentiation contributes to the proliferation and migration of vascular smooth muscle cells

**DOI:** 10.3389/fcell.2022.1003349

**Published:** 2022-10-13

**Authors:** Norihiko Sasaki, Kazumi Hirano, Yuuki Shichi, Yoko Itakura, Toshiyuki Ishiwata, Masashi Toyoda

**Affiliations:** ^1^ Department of Geriatric Medicine (Vascular Medicine), Tokyo Metropolitan Institute of Gerontology, Tokyo, Japan; ^2^ Molecular Neurophysiology Research Group, Biomedical Research Institute, The National Institute of Advanced Industrial Science and Technology (AIST), Ibaraki, Japan; ^3^ Division of Aging and Carcinogenesis, Research Team for Geriatric Pathology, Tokyo Metropolitan Institute of Gerontology, Tokyo, Japan

**Keywords:** smooth muscle cell, phenotype switching, ganglioside, polycomb repressor complex 2, enhancer of zeste homolog 2, proliferation, migration

## Abstract

Phenotypic switching between contractile (differentiated state) and proliferative (dedifferentiated state) vascular smooth muscle cells (VSMCs) is a hallmark of vascular remodeling that contributes to atherosclerotic diseases. Gangliosides, a group of glycosphingolipids, have been detected in atherosclerotic lesions and are suspected to contribute to the disease process. However, the underlying mechanism, specifically with respect to their role in VSMC phenotype switching, is not clear. In this study, we sought to reveal the endogenous expression of gangliosides and their functional significance in VSMCs during atherosclerosis. We found that switching from the contractile to proliferative phenotype was accompanied by upregulation of *a*- and *b*-series gangliosides, which in turn, were regulated by polycomb repressor complex 2 (PRC2). Downregulation of ganglioside expression using an siRNA targeting ST3GAL5, which is required for the synthesis of *a*- and *b*-series gangliosides, attenuated the proliferation and migration of dedifferentiated VSMCs. Therefore, we concluded that the increased expression of *a*- and *b*-series gangliosides *via* PRC2 activity during dedifferentiation is involved in the proliferation and migration of VSMCs. Gangliosides may be an effective target in VSMCs for atherosclerosis prevention and treatment.

## Introduction

Vascular smooth muscle cells (VSMCs) are specialized blood vessel cells that control blood vessel tone, pressure, and flow. In the tunica media of normal adult blood vessels, VSMCs reside in a quiescent state and exhibit a contractile (differentiated) phenotype characterized by the expression of contractile markers, such as alpha smooth muscle actin (α-SMA), smooth muscle 22 alpha (SM22α), and smooth muscle calponin (Gordon et al., 1990). VSMCs retain remarkable phenotypic plasticity and can be regulated in response to various environmental stimuli, including growth factors, mechanical injury, and reactive oxygen species (Zhang et al., 2017; Alencar et al., 2020; Wang et al., 2021; Zhang J. et al., 2021), which leads to marked changes in the VSMC phenotype and behavior. During vessel injury and disease, VSMCs undergo phenotypic switching to a dedifferentiated state. This proliferative phenotype is characterized by decreased expression of contractile proteins and increased expression of non-smooth muscle proteins. The phenotypic switching of VSMCs from a contractile to proliferative phenotype triggers cell migration to the intima, promotes proliferation, and induces the synthesis of extracellular matrix proteins (Gomez and Owens, 2012). The phenotypic modulation of VSMCs is important for vascular injury repair; however, it also plays a major role in the pathogenesis of many diseases, including atherosclerosis (Miano et al., 2021). Therefore, regulation of VSMC phenotype switching is important for maintaining vascular homeostasis. To date, many studies have focused on identifying the molecular mechanisms, including identifying the molecules involved in phenotype switching of VSMCs (Zhang F. et al., 2021); however, many aspects of this phenomenon remain poorly understood.

Gangliosides (Schnaar et al., 2022), a group of acidic glycosphingolipids possessing one or more sialic acid residues on their carbohydrate moieties, are mainly located in sphingolipid and cholesterol-enriched domains called lipid rafts (Sezgin et al., 2017). Under physio-pathological conditions, changes in the expression levels of gangliosides can affect the localization of raft-associated proteins, resulting in reduced membrane fluidity, followed by cellular dysfunction, such as attenuation of cellular signaling (Sonnino et al., 2018; Sasaki et al., 2019b; Zhuo and Guan, 2019). According to the Svennerholm classification, gangliosides can be classified into four series (*o*-, *a*-, *b*-, and *c*-series) based on the number of sialic acid residues (from 0 to 3) linked to the inner galactose residue (Svennerholm, 1980). Both *a*-series (GM3, GM2, GM1, and GD1a) and *b*-series (GD3, GD2, GD1b, and GT1b) gangliosides have been well characterized in several types of tissues and cells, including blood vessels and VSMCs (Cavdarli et al., 2019; Sasaki and Toyoda, 2019). GM3 accumulates in atherosclerotic lesions (Bobryshev et al., 2006). Tumor necrosis factor (TNF)-induced proliferation and induction of matrix metalloproteinase (MMP)-9, which is implicated in the progression of atherosclerotic lesions (Park et al., 2011), is inhibited upon GM3 overexpression by GM3 synthase gene transfection in mouse VSMCs. In this study, treatment with anti-GM3 antibodies reversed the inhibitory effects of GM3, indicating that GM3 controls the proliferation and migration of VSMCs during the formation of atherosclerotic lesions. In experiments using rat aortic VSMCs, exogenous addition of GM1 and GM2, but not GM3, induced the activation of the extracellular signal-regulated kinase pathway and promoted VSMC proliferation (Gouni-Berthold et al., 2001). In addition, increased GD3 levels are associated with atherosclerosis (Chatterjee et al., 1997). Exogenous addition of GD3 has a dual role in modulating the proliferation and apoptosis of human VSMCs (Bhunia et al., 2002), in which low concentrations of GD3 promote proliferation, whereas high concentrations induce apoptosis. GD3 overexpression *via* the GD3 synthase (*ST8SIA1*) gene transfection attenuates platelet-derived growth factor (PDGF)-induced activation of the extracellular signal-regulated kinase (ERK) pathway and suppresses the proliferation of mouse VSMCs (Moon et al., 2004). Furthermore, GD3 overexpression inhibited TNFα-induced MMP-9 induction (Moon et al., 2004).

The above-described published studies have demonstrated an association of VSMC functions with pathological conditions, such as arteriosclerosis, and have suggested a potential importance of gangliosides in this regard. However, these studies have mostly relied on exogenous addition of gangliosides or their overexpression in VSMCs *via* ganglioside synthase transfection, while the mechanism of endogenous expression of gangliosides and their role in differentiation and dedifferentiation of VSMCs during physio/pathological conditions have not yet been elucidated. The aim of this study was to investigate the endogenous expression of gangliosides and their functional significance in the phenotype switching of VSMCs.

## Materials and methods

### Growth factors and chemicals

The enhancer of zeste homolog 2 (EZH2) inhibitor, GSK126 (Selleck Chemicals, Houston, TX, United States), was reconstituted in DMSO and used at a final concentration of 10 µM in culture medium. The same dilution of DMSO was used as vehicle control. For the incubation of cells with exogenous GM1, 5 µM GM1 (Sigma-Aldrich, St. Louis, MO, United States) in culture medium was used.

### Cell culture

Human aortic smooth muscle cells (HASMCs) were purchased from Lonza (Walkersville, MD, United States). HASMCs were grown in HASMC growth media [SmGM™-2 (Lonza) containing supplements and growth factors, including FGF-2 and EGF, in the kit (Lonza)]. HASMC cultures between passages two and five were used in all experiments. HASMCs were incubated with SmGM™-2 supplemented with 1% FBS for 7 days for differentiation.

### Transfection of siRNA targeting ST3GAL5

Cells were plated at a density of 2 × 10^5^ cells into 35-mm dishes and transfected 24 h later with 5 nM siRNAs (Silencer^®^ Select; Thermo Fisher Scientific, Waltham, MA, United States) targeting *ST3GAL5* (si-1: 5-CGA UGU GAU AAG GUU ATT-3; si-2: 5-CCA GCU UGU UAU UAA AAG ATT-3), or Silencer negative control siRNA (siCont) using Lipofectamine™ RNAiMAX Transfection Reagent (Thermo Fisher Scientific) according to the manufacturer’s protocol.

### Scanning electron microscope analysis

Cells were fixed for 30 min with 2.5% glutaraldehyde in 0.1 M phosphate buffer (pH 7.4). The glutaraldehyde solution was then removed, and the cells were washed with phosphate buffered saline (PBS). The cells were post-fixed with osmium tetroxide for 30 min to prevent the cells from collapsing during sample preparation. After complete dehydration *via* a graded ethanol series, cell samples were suspended in 100% ethanol, air-dried, and coated with a platinum layer using an MSP-1S sputter coater (Shinku Device, Ibaraki, Japan). Cells were examined and micrographed using a Phenom Pro desktop scanning electron microscope (SEM) with secondary electrons (Thermo Fisher Scientific). Spherical cultures were prepared in triplicate for SEM observation.

### Cell cycle assay

Cells were washed in PBS, resuspended, and stained with cell cycle assay solution (deep red; Dojindo Molecular Technologies, Inc, Rockville, MD, United States) at 37°C for 15 min. Cell cycle profiles were obtained using a FACSAria™ cell sorter (Becton Dickinson, Franklin Lakes, NJ, United States) at 640 nm. Data were analyzed using the FlowJo software (Becton Dickinson).

### Migration assay

Cell culture inserts (8- µm pore size and 6-mm diameter; Corning, Glendale, AZ, United States) were used according to the manufacturer’s instructions. HASMC growth media was placed in the lower chamber of a 24-well plate. Cells were plated at a density of 1 × 10^5^ cells/500 µL SmGM™-2 without supplements on the upper surface of the inserts. Following 6 h of incubation at 37°C, cells that had migrated through the membrane to the lower surface of the filter were fixed, stained with a Diff-Quik staining kit (Polysciences, Inc, Warrington, PA, United States), and then imaged using Mantra, multi-spectral microscopy. The images were loaded into the inForm software ver. 2.4 (Perkin-Elmer, Inc, Waltham, MA, United States) to establish the number of migrated or invaded cells in 12 random fields at ×20 magnification.

### Real-time PCR

Total RNA was isolated from cells using the RNeasy Plus Mini Kit (QIAGEN, Hilden, Germany) and subsequently reverse-transcribed using the ReverTra Ace^®^ qPCR RT Kit (Toyobo, Osaka, Japan). Real-time PCR (RT-PCR) was performed using the Power SYBR^®^ Green kit (Applied Biosystems, Foster City, CA, United States) and StepOnePlus™ real-time PCR system (Applied Biosystems). β-ACTIN was used as an internal control. The threshold crossing value for each transcript was normalized to that of the internal control. Relative quantification of each mRNA was performed using the comparative Ct method. The primer sets used for RT-PCR are listed in Supplementary Table S1.

### Fluorescence-activated cell sorting analysis

As previously described (Sasaki et al., 2015), cells were harvested with Accutase^®^ cell detachment solution (Merck Millipore, Billerica, MA, United States), and dissociated single cells were incubated with primary antibodies diluted in fluorescent activated cell sorting (FACS) buffer (0.5% [w/v] bovine serum albumin [BSA] and 0.1% [w/v] sodium azide in PBS) for 30 min on ice. After washing, the cell suspension was incubated with Alexa Fluor^®^ 488-conjugated secondary antibodies (Molecular Probes, Eugene, OR, United States) and diluted in FACS buffer for 30 min on ice. For the detection of GM1 or *c*-series gangliosides, cells were incubated with Alexa Fluor^®^ 647-conjugated cholera toxin B subunit (Molecular Probes) or APC-conjugated anti-A2B5 antibody (Miltenyi Biotec, Bergisch Gladbach, Germany) diluted in FACS buffer for 30 min on ice, respectively. Cell sorting and analysis were performed using a FACSAria™ Cell Sorter. The following primary antibodies were used: anti-GM3 (NBT Laboratories Inc, Tokyo, Japan), anti-GM2 (TCI, Tokyo, Japan), anti-GD1a (TCI), anti-GT1b (TCI), anti-GD3 (Merck Millipore), anti-GD2 (TCI), and anti-GD1b (TCI). Mean fluorescence intensity was calculated by subtracting the intensity of the control.

### Chromatin immunoprecipitation analysis

Chromatin immunoprecipitation (Chip) was performed according to a protocol published by Agilent Technologies (Santa Clara, CA, United States), with the following modifications. Briefly, the cells were fixed with a 11% formaldehyde solution for 10 min. After harvesting the fixed cells, they were lysed in lysis buffer and sonicated using a Misonix XL2020 sonicator (MISONIX Inc, NY, http://misonix.com) until the DNA fragments were 200–600 bp in length and 3.0% of the total volume was stored as input at -20°C until use. Immunoprecipitation was performed at 4°C overnight with anti-H3K27me3 (ab6002; Abcam, Cambridge, UK). DNA/beads were washed once with a low salt buffer (20 mM Tris-HCl pH 7.4, 150 mM NaCl, 2 mM EDTA, 0.1% sodium dodecyl sulfate [SDS], 1% Triton X-100), and then further washed once with a high-salt buffer (20 mM Tris-HCl pH 7.4, 400 mM NaCl, 2 mM EDTA, 0.1% SDS, 1% Triton X-100) before washing with radioimmunoprecipitation assay (RIPA) buffer. Immune complexes were disrupted with direct elution buffer (50 mM Tris-HCl pH8.0, 10 mM EDTA, 1% SDS), and the covalent links between immunoprecipitates and input chromatin were disrupted by incubation at 65°C overnight. DNA was further incubated with RNase A and proteinase K (Nacalai Tesque, Kyoto, Japan), purified by phenol extraction, and precipitated with ethanol. DNA pellets were dissolved in Tris-EDTA buffer (10 mM Tris-HCl, 1 mM EDTA pH8.0). The co-immunoprecipitated DNA was detected by quantitative PCR (qPCR) using primers for the *ST8SIA1* gene (Supplementary Table S2).

### Immunostaining

Cells were fixed with 4% (w/v) paraformaldehyde and then washed. Subsequently, cells were permeabilized with 0.1% (v/v) Triton™ X-100 and blocked with PBS containing 1% (w/v) BSA and 5% (v/v) normal goat serum. Following washing, the cells were incubated with anti-GD1a and monoclonal mouse anti-α-SMA (ab7817; Abcam) anti-bodies at 4°C overnight. The cells were washed, stained with Alexa Fluor^®^ 488- and Alexa Fluor^®^ 546-conjugated secondary antibodies (Molecular Probes), and counterstained with 4ʹ,6-diamidino-2-phenylindole (DAPI). Immunofluorescence images were acquired using a confocal laser scanning microscope (Leica Microsystems, Wetzlar, Germany).

### Immunoblotting

Cells were lysed with lysis buffer (50 mM Tris-HCl pH 7.4, 150 mM NaCl, 1.5 mM MgCl_2_, 5 mM EDTA, and 1% Triton™ X-100) containing protease and phosphatase inhibitor cocktails. To study downstream signaling in response to HASMC growth media, cells were incubated with freshly changed HASMC growth media for 5 min. The cell lysates were separated by SDS-PAGE and then transferred onto polyvinylidene difluoride (PVDF) membranes (Merck Millipore). After blocking, the membranes were incubated with the following primary antibodies: mouse monoclonal anti-H3K27me3 (ab6002; Abcam), polyclonal rabbit anti-Histone H3 (#9715; Cell Signaling, Danvers Technology, MA, USA), monoclonal mouse anti-α-SMA (ab7817; Abcam), polyclonal rabbit anti-SM22α (ab14106; Abcam), polyclonal rabbit anti-ST3GAL5 (14614-1-AP; Proteintech Group, Inc., Rosemont, IL, United States), polyclonal rabbit anti-ST8SIA1 (24918-1-AP; Proteintech Group), monoclonal mouse anti-EZH2 (sc-137255; Santa Cruz Biotechnology, Dallas, TX, USA), monoclonal rabbit anti-ERK1/2 (#4695; Cell Signaling), monoclonal rabbit anti-p-ERK1/2 (#4370; Cell Signaling), monoclonal rabbit anti-focal adhesion kinase (FAK) (ab40794; Abcam), monoclonal rabbit anti-p-FAK (ab81298; Abcam), and monoclonal mouse anti-β-actin (A5316; Sig-ma-Aldrich). The membranes were then incubated with the appropriate peroxidase-conjugated secondary antibodies (Cell Signaling Technology), washed, and developed with ECL™ Prime reagents (GE Healthcare, Piscataway, NJ, United States).

### Statistical analysis

Results are expressed as the mean ± SD of three independent experiments. Statistical analyses were performed using Microsoft Excel for Mac statistical analysis, version 3.0. Unpaired Student’s *t*-test and one-way ANOVA with Tukey’s honestly significant difference (HSD) test were used to compare two or more groups, respectively.

## Results

### *a*- and *b*-series ganglioside expression levels in differentiated VSMCs are lower than those in proliferative VSMCs

**FIGURE 1:**
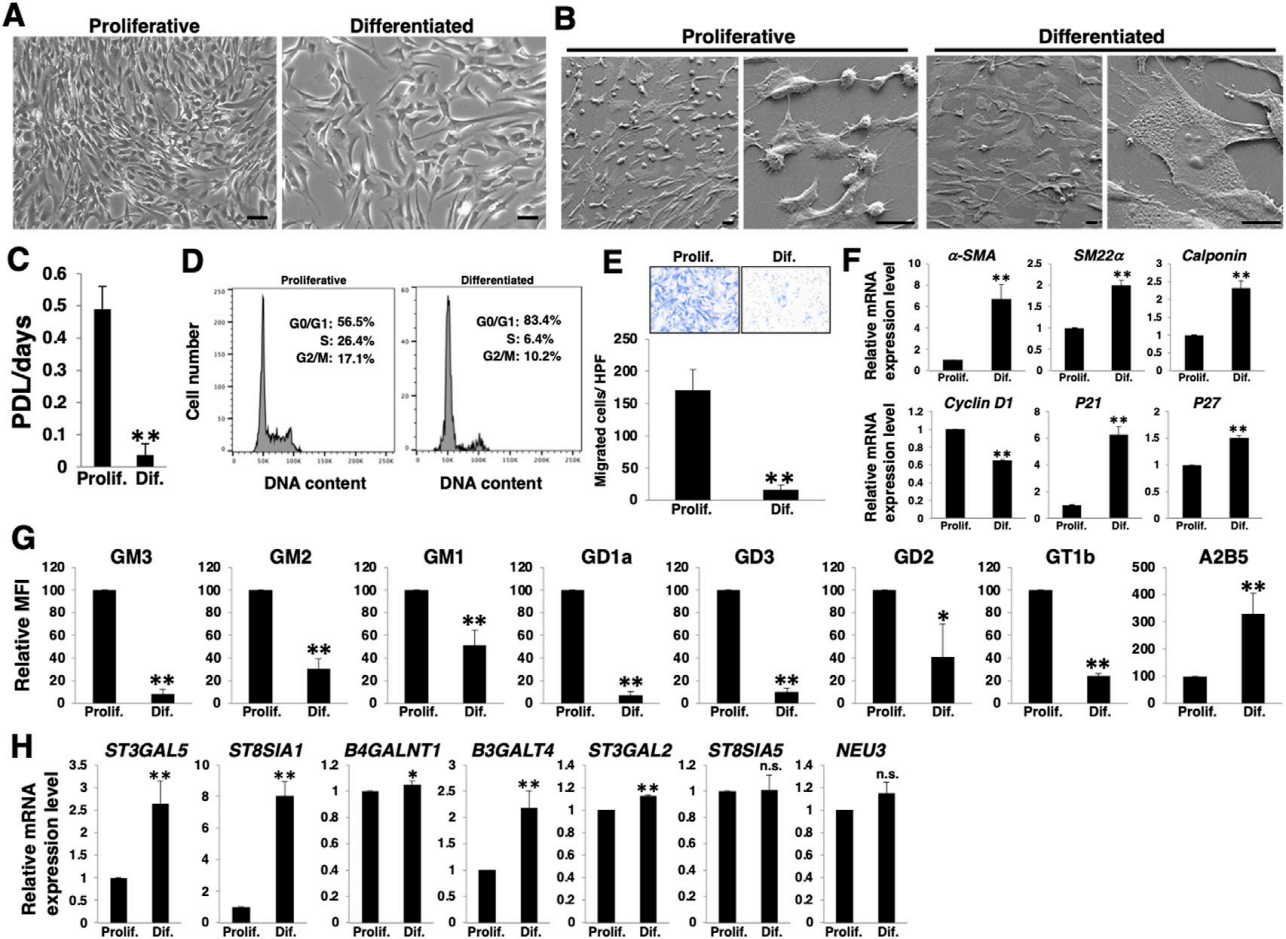
Expression levels of *a*- and *b*-series gangliosides in differentiated and proliferative vascular smooth muscle cells (VSMCs). Human aortic smooth muscle cells (HASMCs) were cultured under normal and low serum conditions for 7 days to obtain proliferative and differentiated cells, respectively. The cells were harvested and then used for the following experiments. **(A)** Representative phase contrast images of the cells. Scale bar: 2 µm. **(B)** Representative scanning electron microscope (SEM) images of the cells. Scale bar: 20 µm. **(C)** Cell growth rates are expressed as population doubling level (PDL)/days. Data are presented as mean ± standard deviation (SD) of three independent experiments. **(D)** Cell cycle analysis was performed as described in the Materials and Methods. Representative results of one out of three experiments are shown. **(E)** Migration capacity of the cells, expressed as the number of cells migrating across the culture insert in each high-power field (HPF). Representative results from measurements of 12 fields are shown. **(F)** Real-time PCR (RT-PCR) analysis of contractile or growth-related genes using cDNA derived from the cells. The results are shown as fold change relative to values in proliferative cells, which are normalized to 1. Data are expressed as means ± SD of three independent experiments. **(G)** Flow cytometry data indicating the level of ganglioside expression, determined as mean fluorescence intensities (MFI). Values in the differentiated cells are expressed as a percentage of corresponding values in proliferative cells, which were normalized to 100%. Data are expressed as means ± SD of three independent experiments. **(H)** RT-PCR analysis of the glycosyltransferases shown in Supplementary Figure S1 and *NEU3* using cDNA derived from the cells. The results are shown as fold change relative to values for proliferative cells, which are normalized to 1. Data are expressed as means ± SD of three independent experiments. *p < 0.05; **p < 0.01, n. s, not significant; Prolif, proliferative; Dif, differentiated.

To clarify the phenotypic plasticity of VSMCs, we cultured primary HASMCs under normal culture and low serum conditions, as described in the Materials and Methods section and examined for cell proliferation, migration ability, and contractile markers. When cultured in low serum medium, HASMCs differentiate with increased expression of contractile markers (Chen et al., 2016). As expected, HASMCs cultured under the low serum (1% FBS) condition for 7 days exhibited an enlarged and flattened morphology (Figures 1A,B), low growth rate (Figure 1C), and cell cycle arrest in the G0/G1 phase (Figure 1D). Furthermore, these cells showed lower migration ability than proliferative cells (Figure 1E). RT-PCR analysis showed an increase in the expression of smooth muscle contractile markers (*α-SMA*, *SM22α*, and *calponin*) and cell cycle inhibitors (*p21* and *p27*) with a concomitant decrease in the proliferative marker *cyclin D1* in HASMCs cultured under low serum conditions (Figure 1F), indicating a differentiation induction.

We then examined the cell surface expression of gangliosides in HASMCs at a differentiated (cultured under low serum conditions) or proliferative (under normal conditions) state. FACS analysis revealed that nearly all *a*- and *b*-series gangliosides examined (Supplementary Figure S1) were downregulated in the differentiated state (Figure 1G; Supplementary Figure S2A). In contrast, *c*-series gangliosides, which are known as A2B5 antigens (Saito et al., 2001) (Supplementary Figure S1), were upregulated in the differentiated state (Figure 1G; Supplementary Figure S2A). To elucidate the mechanisms contributing to the observed changes in ganglioside levels upon differentiation, we analyzed the expression levels of the glycosyltransferases involved in the ganglioside synthesis pathways (Supplementary Figure S1) and sialidase (NEU3), which modulates ganglioside content by removing sialic acid (Sasaki et al., 2003). RT-PCR analysis revealed that the expression of *ST3GAL5*, *ST8SIA1*, and *B3GALT4* was particularly increased in the differentiated state of HASMCs (Figure 1H). The results of the increased expression of ST3GAL5, which synthesizes ganglioside GM3 and contributes to the synthesis of *a*-, *b*-, and *c*-series gangliosides (Supplementary Figure S1), suggest that the synthesis of *a*-, *b*-, and *c*-series gangliosides in glycosphingolipids on the plasma membrane increased during differentiation. However, as shown in Figure 1G, *a*- and *b*-series gangliosides were downregulated. ST8SIA1 is involved in the branching between *a*- and *b*-/*b*- and *c*-series gangliosides (Supplementary Figure S1). Hence, it was suggested that the marked upregulation of ST8SIA1 served an advantage for the synthesis of *c*-series gangliosides, more so than the synthesis of *a*- and *b*-series gangliosides, resulting in a relative downregulation of the ratio of *a*- and *b*-series gangliosides but an upregulation of that of *c*-series ganglioside.

### *a*- and *b*-series gangliosides expression levels increase with VSMCs dedifferentiation

**FIGURE 2:**
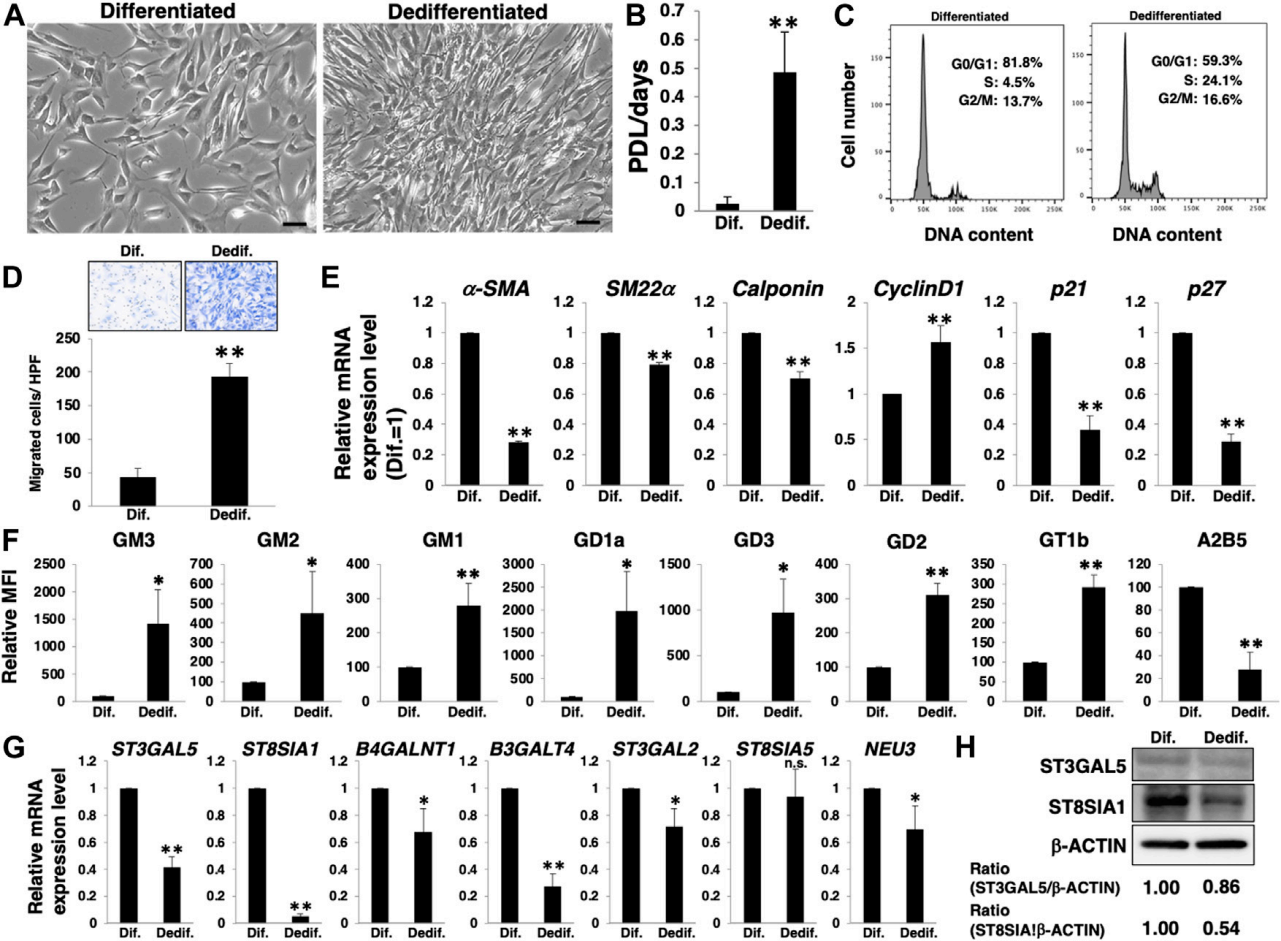
Expression levels of *a*- and *b*-series gangliosides increase with the dedifferentiation of vascular smooth muscle cells (VSMCs). Differentiated human aortic smooth muscle cells (HASMCs) were re-cultured under normal or low serum conditions for 3 days. The cells were then harvested and used for subsequent experiments. **(A)** Representative phase contrast images of the re-cultured cells. Scale bar: 2 µm. **(B)** Growth rates are expressed as population doubling level (PDL)/days. Data are expressed as mean ± standard deviation (SD) of three independent experiments. **(C)** Representative cell cycle analysis data from one out of three experiments. **(D)** Migration capacity of the cells, expressed as the number of cells migrating across the culture insert in each high-power field (HPF). **(E)** Real-time PCR (RT-PCR) analysis of contractile or growth-related genes using cDNA derived from the cells. The results are shown as fold change relative to values in differentiated cells, which are normalized to 1. Data are expressed as means ± SD of three independent experiments. **(F)** Flow cytometry data showing the level of ganglioside expression, determined as mean fluorescence intensities (MFI). Values in the dedifferentiated cells are expressed as a percentage of corresponding values in differentiated cells, which were normalized to 100%. Data are expressed as means ± SD of three independent experiments. **(G)** RT-PCR analysis of the glycosyltransferases shown in Supplementary Figure S1 and *NEU3* using cDNA derived from the cells. The results are shown as fold change relative to the corresponding values in differentiated cells, which are normalized to 1. The values shown are the means ± SD of three independent experiments. **(H)** Immunoblotting for ST3GAL5, ST8SIA1, and β-ACTIN in the cells. Densitometric value ratios are shown below the respective protein band images (Dif. = 1.00). *p < 0.05; **p < 0.01, Dif, differentiated, Dedif, dedifferentiated.

To determine whether the differentiation of HASMCs and the associated molecular profile, as described in Figure 1, were reversible, we re-cultured differentiated HASMCs under normal culture conditions. Three days after re-culture, HASMCs exhibited a higher density than dedifferentiated cells (Figure 2A). Furthermore, re-cultured HASMCs showed a higher growth rate (Figure 2B) with activation of the cell cycle (Figure 2C) and higher migration rate than dedifferentiated cells (Figure 2D). RT-PCR analysis indicated a decrease in the expression of smooth muscle contractile markers (*α-SMA*, *SM22α*, and *calponin*), cell cycle inhibitors (*p21* and *p27*), and an increase in the proliferative marker *cyclin D1* in re-cultured HASMCs (Figure 2E). These results indicate that dedifferentiation was induced in HASMCs. We then examined the expression levels of gangliosides and related genes. FACS analysis revealed that all the examined *a*- and *b*-series gangliosides were upregulated along with downregulation of *c*-series gangliosides (Figure 2F;Supplementary Figure S1B). Moreover, the mRNA expression of nearly all examined glycosyltransferases, as well as *NEU3,* was downregulated in the dedifferentiated state (Figure 2G). The ST3GAL5 downregulation (Figures 2G,H) indicated that the synthesis of gangliosides in glycosphingolipids on the plasma membrane decreased during HASMCs dedifferentiation. Furthermore, the marked decline of ST8SIA1 expression (Figures 2G,H) was expected to lead to a relative upregulation of the ratio of *a*- and *b*-series gangliosides, but rather induced a relative downregulation of *c*-series gangliosides. Figures 1, 2 show that the phenotype switching of VSMCs from the contractile (differentiated state) to proliferative (dedifferentiated state) phenotype was accompanied by a relative upregulation of *a*- and *b*-series gangliosides and downregulation of *c*-series gangliosides in accordance with the glycosyltransferase expression.

### Polycomb repressor complex 2, including EZH2, is involved in *ST8SIA1* expression during VSMC dedifferentiation

**FIGURE 3:**
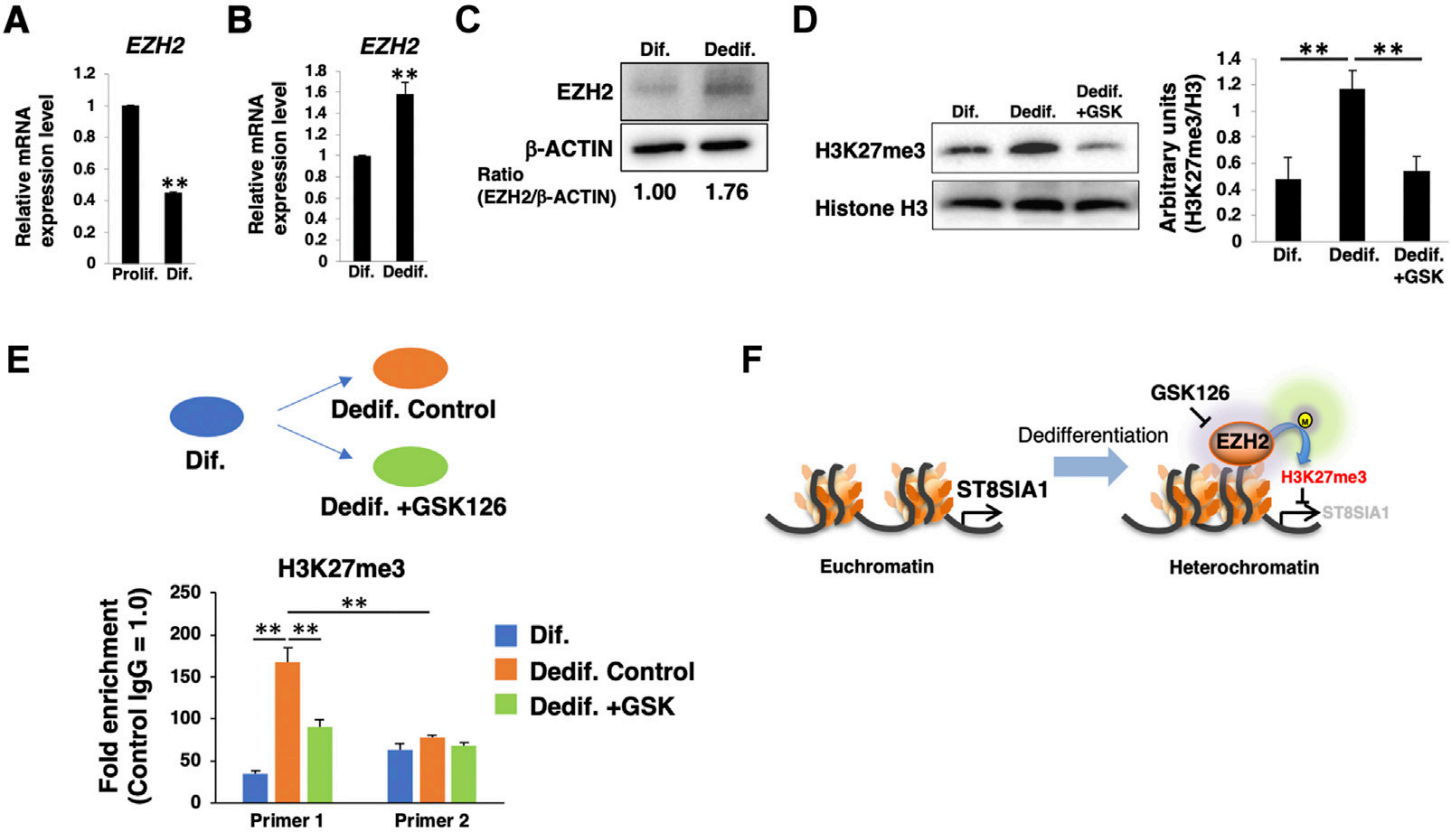
PRC2 is involved in dedifferentiation of vascular smooth muscle cells (VSMCs). **(A)** Real-time PCR (RT-PCR) analysis of *EZH2* using cDNA derived from human aortic smooth muscle cells (HASMCs) cultured for 7 days under normal or low serum conditions to obtain proliferative and differentiated cells, respectively. The results are shown after normalization against the values obtained for cells cultured under normal conditions (value = 1). The data are expressed as means ± standard deviation (SD) of three independent experiments. **(B)** RT-PCR analysis of *EZH2* using cDNA derived from differentiated HASMCs re-cultured for 3 days under normal or low serum conditions. The results are shown after normalization against the values obtained for cells cultured under low serum conditions (value = 1). Data are expressed as means ± SD of three independent experiments. **(C)** Immunoblotting for EZH2 and β-ACTIN in the cells. Densitometric value ratios are shown below the respective protein band images (Dif. = 1.00). **(D)** Western blot analysis of H3K27me3 expression in differentiated HASMCs re-cultured under normal conditions with or without 10 µM GSK126 for 3 days. The histograms show mean densitometric readings ±SD for H3K27me3 normalized to the loading controls (Histone H3). Results are expressed as means ± SD of three independent experiments. **(E)** Chromatin immunoprecipitation assays for differentiated HASMCs 3 days after re-culture with or without 10 µM GSK126 were performed using anti-H3K27me3 antibody and control IgG, followed by RT-PCR with the primer sets amplifying the proximity of the *ST8SIA1* gene, as described in the Materials and Methods. The graph shows the representative relative amounts (value ±SD normalized to IgG control) from two independent experiments. **(F)** Summary of epigenetic regulation through EZH2 activity during dedifferentiation of VSMCs. *p < 0.05; **p < 0.01, Prolif, proliferative, Dif, differentiated, Dedif, dedifferentiated.

The ganglioside expression levels seem to be mainly regulated by glycosyltransferases in the proliferative/dedifferentiated and differentiated states of HASMCs. Among the glycosyltransferases involved in ganglioside synthesis pathways (Supplementary Figure S1), ST8SIA1 is the key enzyme that controls the biosynthesis of *a*-, *b*-, and *c*-series gangliosides, and suggests that the expression levels of ST8SIA1 affect the relative expression levels of *a*-, *b*-, and *c*-series gangliosides in HASMCs. EZH2 directly binds to promotor regions of *ST8SIA1*, and inhibition of EZH2 enhances the expression of this gene in Ewing sarcoma cells (Kailayangiri et al., 2019). EZH2 is the catalytic component of PRC2, which catalyzes tri-methylation of histone H3 at Lys 27 (H3K27me3) to negatively regulate gene expression (Simon and Lange, 2008). Accumulating evidence led us to hypothesize that PRC2, including EZH2, is involved in the expression of *ST8SIA1* in HASMCs. To test this hypothesis, we first examined the expression levels of EZH2 between the proliferated/dedifferentiated and differentiated states. During the differentiated state of HASMCs, *EZH2* expression was decreased compared to that in the proliferative state (Figure 3A) but was increased at both mRNA and protein levels by dedifferentiation induction (Figures 3B,C). We then examined H3K27me3 level to confirm the effect of EZH2. In dedifferentiated HASMCs, 10 µM GSK126 (the EZH2 highly selective inhibitor) (McCabe et al., 2012), worked well for reduction of H3K27me3 (Supplementary Figure S3A). Indeed, we confirmed that H3K27me3 level was upregulated during dedifferentiation and inhibited by GSK126 treatment (Figure 3D; Supplementary Figure S3B). These results suggested that EZH2 affects gene expression during dedifferentiation *via* H3K27me3 regulation. Deposited datasets in the ChIP-atlas (Oki et al., 2018), a database of ChIP-seq (http://chip-atlas.org/), showed that EZH2 (SRX1998,402) and H3K27me3 (SRX1998,407) are enriched close to the transcription initiation site (TSS) of *ST8SIA1* in rhabdomyosarcoma (Supplementary Figure S3C), suggesting that EZH2 can control *ST8SIA1* expression. In contrast to cancer cells, this regulation mechanism has not yet been reported in normal cells. To determine whether EZH2 catalyzes H3K27me3 at the proximity region of *ST8SIA1* in HASMCs, we designed primer sets for detection of the predicted EZH2-regulated region or irrelevance region, respectively (primers one and two in Supplementary Figure S3C) and performed ChIP-qPCR analysis. As shown in Figure 3E, the level of H3K27me3 enrichment detected by primer one was higher than that by primer two in the dedifferentiated state, indicating that the designed primers worked in this genomic region. We then confirmed the upregulation of H3K27me3 enrichment at the predicted EZH2-regulated region (detected by primer 1) near the *ST8SIA1* gene in dedifferentiated cells compared with differentiated cells, but this increase was attenuated by treatment with GSK126, indicating that the *ST8SIA1* gene is controllable *via* EZH2 in HASMCs. Altogether, these results suggest that PRC2, including EZH2, regulates H3K27me3 at the promotor region of *ST8SIA1* and downregulates its expression during the dedifferentiation of VSMCs (Figure 3F).

### PRC2 contributes to the gangliosides expression during VSMCs dedifferentiation

**FIGURE 4:**
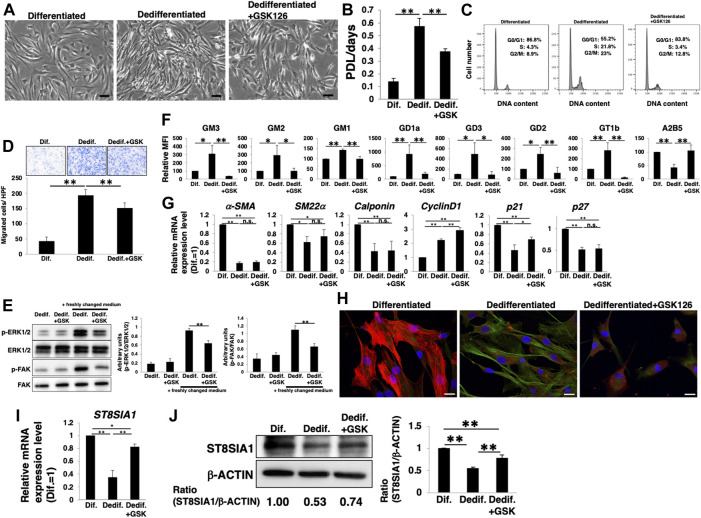
PRC2 contributes to the expression of gangliosides during dedifferentiation of vascular smooth muscle cells (VSMCs). Differentiated human aortic smooth muscle cells (HASMCs) were cultured under low serum conditions or re-cultured under normal conditions with or without 10 µM GSK126 for 3 days. The cells were harvested and used in subsequent experiments. **(A)** Representative phase contrast images of the cells. Scale bar: 2 µm. **(B)** Cell growth rates are expressed as population doubling level (PDL)/days. Data are expressed as mean ± standard deviation (SD) of three independent experiments. **(C)** Representative cell cycle analysis data from one out of three experiments. **(D)** Migration capacity of the cells, expressed as the number of cells migrating across the culture insert in each high-power field (HPF). **(E)** Immunoblotting image showing the expression of p-ERK1/2, ERK1/2, p-FAK, and FAK. The histograms show densitometric readings for the phosphorylated proteins normalized to those of the loading controls. Results are expressed as means ± SD of three independent experiments. **(F)** Flow cytometry data showing the level of ganglioside expression, determined as mean fluorescence intensities (MFI). Values in the differentiated cells are expressed as a percentage of corresponding values in differentiated cells, which were normalized to 100%. Data are expressed as means ± SD of three independent experiments. **(G)** Real-time PCR (RT-PCR) analysis of contractile or growth-related genes using cDNA derived from the cells. The results are shown as fold change relative to values in differentiated cells, which are normalized to 1. Data are expressed as means ± SD of three independent experiments. **(H)** Immunocytochemical staining performed in the cells. Representative images are shown (α-SMA, red; GD1a, green; DAPI, blue). Scale bar: 20 µm. **(I)** RT-PCR analysis of *ST8SIA1* using cDNA derived from the cells. The results are shown as fold change relative to corresponding values for differentiated cells, which are normalized to 1. Data are expressed as means ± SD of three independent experiments. **(J)** Immunoblotting for ST8SIA1 and β-ACTIN in the cells. Ratio of densitometric values is shown below the respective protein band images (Dif. = 1.00). The histograms show mean densitometric readings ±SD for ST8SIA1 normalized to the loading controls (β-ACTIN). Results are expressed as means ± SD of three independent experiments. *p < 0.05; **p < 0.01, n. s, not significant; Dif, differentiated,; Dedif, dedifferentiated.

Next, we examined whether the activation of PRC2, including EZH2, leads to a relative upregulation of *a*- and *b*-series gangliosides and contributes to the phenotype switching of HASMCs from a contractile (differentiated state) to proliferative (dedifferentiated state) phenotype. Dedifferentiation of differentiated HASMCs was accompanied with increased cell density (Figure 4A), proliferation (Figure 4B), cell cycle activation (Figure 4C), and migration (Figure 4D), all of which were attenuated by GSK126 treatment (Figures 4A‒D). We further examined downstream molecules of proliferation- and migration-related signaling in GSK126-treated HASMCs. Western blotting showed that the phosphorylation levels of ERK (proliferation-related) and FAK (migration-related) were reduced in GSK126-treated HASMCs compared with those in non-treated cells (Figure 4E), indicating that proliferation- and migration-related signaling was attenuated by GSK126 treatment. The *a*- and *b*-series ganglioside expression levels, which were relatively increased with dedifferentiation, were comparable to those in differentiated cells following GSK126 treatment (Figure 4F; Supplementary Figure S2C). In contrast, GSK126 treatment did not restore the decreased expression of the markers (*α-SMA*, *SM22α*, *calponin*, and *p27*) observed during dedifferentiation (Figure 4G). Furthermore, the protein levels of smooth muscle contractile markers (α-SMA and SM22α) in GSK126-treated cells were comparable to those in non-treated cells (Supplementary Figure S4). Immunocytochemical staining demonstrated that the expression of the contractile marker, α-SMA, was downregulated in dedifferentiated cells, accompanied with a relative upregulation of GD1a (an *a*-series gangliosides) (Figure 4H left and center); in contrast, in GSK126-treated dedifferentiated cells, level of α-SMA was reduced and that of GD1a was not upregulated (Figure 4H right). These results suggest that dedifferentiation of VSMCs is not affected by attenuation of the increase in *a*- and *b*-series ganglioside levels. The ST8SIA1 expression was maintained during dedifferentiation by GSK126 treatment (Figures 4I,J), suggesting that ST8SIA1 expression may sustain relatively lower levels of *a*- and *b*-series gangliosides. Taken together, these results indicate that PRC2 containing EZH2 is involved in the relative upregulation of *a*- and *b*-series gangliosides during the dedifferentiation of VSMCs, thus affecting proliferation and migration capability.

### The *a*- and *b*-series gangliosides are involved in VSMCs proliferation and migration during dedifferentiation

**FIGURE 5:**
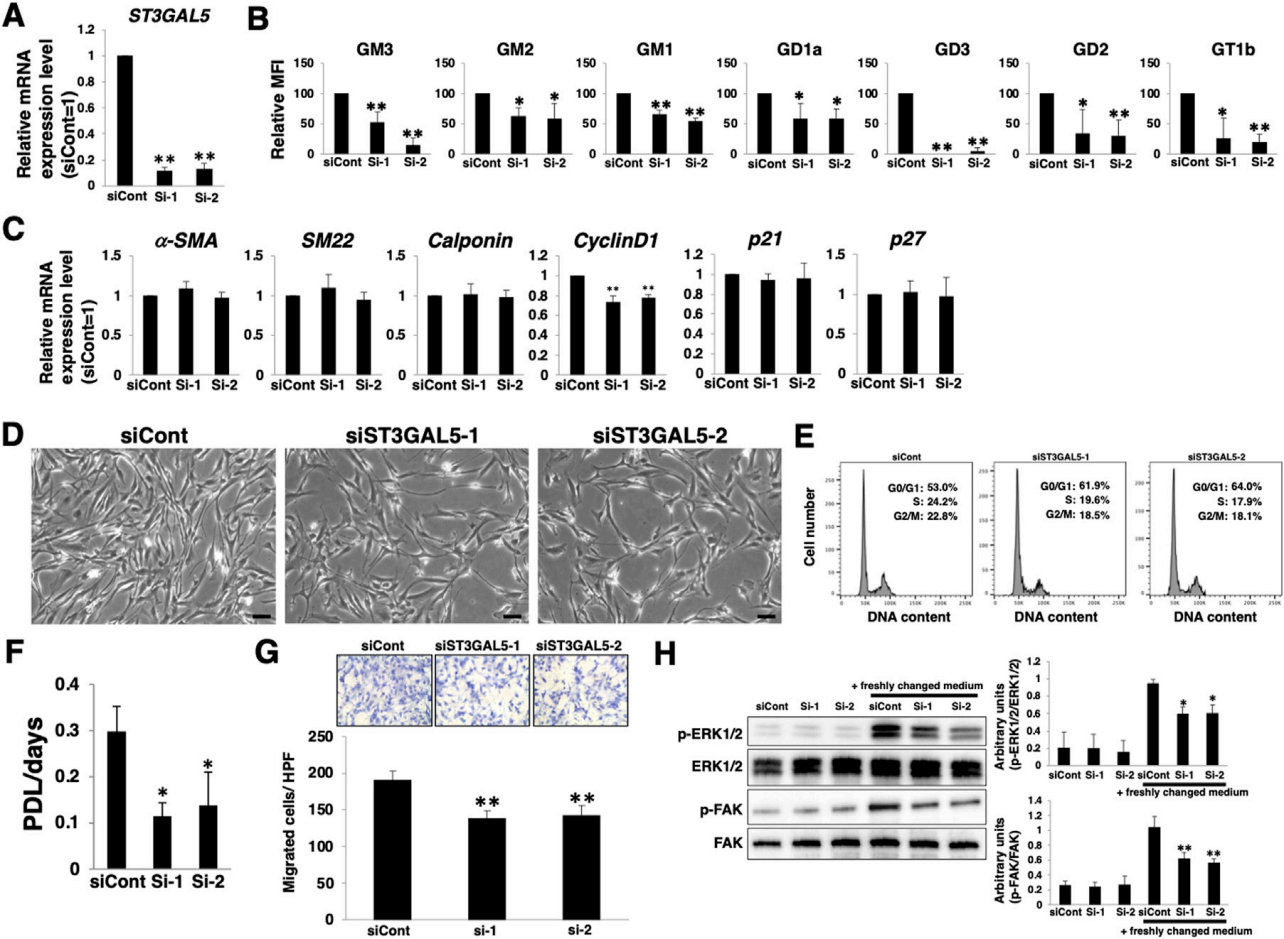
Increase in gangliosides in vascular smooth muscle cells (VSMCs) during dedifferentiation contributes to proliferation and migration. Differentiated human aortic smooth muscle cells (HASMCs) were transfected with siRNA targeting ST3GAL5 and re-cultured under normal conditions for 3 days. The cells were harvested and used in the following experiments. **(A)** Real-time PCR (RT-PCR) analysis of *ST3GAL5* expression using cDNA derived from the cells. The results are shown as fold change relative to values in siCont-transfected cells, which are normalized to 1. Data are expressed as means ± standard deviation (SD) of three independent experiments. **(B)** Flow cytometry data showing the level of ganglioside expression, determined as mean fluorescence intensities (MFI). Values in the cells are expressed as a percentage of corresponding values in siCont-transfected cells, which were normalized to 100%. Data are expressed as means ± SD of three independent experiments. **(C)** RT-PCR analysis of contractile or growth-related genes using cDNA derived from the cells. The results are shown as fold change relative to values in siCont-transfected cells, which are normalized to 1. Data are expressed as means ± SD of three independent experiments. **(D)** Representative phase contrast images of the cells. Scale bar: 2 µm. **(E)** Representative cell cycle analysis data from one out of two independent experiments. **(F)** Cell growth rates are expressed as population doubling level (PDL)/days. Data are expressed as mean ± SD of three independent experiments. **(G)** Migration capacity of the cells, expressed as the number of cells migrating across the culture insert in each high-power field (HPF). **(H)** Immunoblotting image showing the expression of p-ERK1/2, ERK1/2, p-FAK, and FAK. The histograms show densitometric readings for the phosphorylated proteins normalized to those of the loading controls. Results are expressed as means ± SD of three independent experiments. (*p < 0.05, **p < 0.01 vs. siCont-transfected cells). n. s, not significant; Dif, differentiated; Dedif, dedifferentiated.

To clarify whether the relative upregulation of *a*- and *b*-series gangliosides with dedifferentiation is involved in the proliferation and migration of VSMCs, we knocked down *ST3GAL5*. *ST3GAL5* encodes a sialyltransferase that synthesizes ganglioside GM3 and contributes to the synthesis of *a*- and *b*-series gangliosides (Supplementary Figure S1). siRNA targeting *ST3GAL5*-transfected HASMCs reduced *ST3GAL5* mRNA expression (Figure 5A). FACS analysis confirmed that the expression of *a*- and *b*-series gangliosides was reduced in *ST3GAL5*-siRNA-transfected HASMCs (Figure 5B; Supplementary Figure S2D). *a*- and *b*-series ganglioside suppression had no effect on smooth muscle contractile markers (*α-SMA*, *SM22α*, and *calponin*) and the cell cycle inhibitors (*p21* and *p27*), except for a slight reduction in *cyclinD1* expression (Figure 5C). In contrast, cell density, cell cycle, proliferation, and migration were attenuated in *ST3GAL5*-siRNA-transfected HASMCs compared with those in siCont-transfected cells (Figure 5D‒G), indicating that *a*- and *b*-series gangliosides are involved in proliferation and migration. Furthermore, we examined downstream molecules of proliferation- and migration-related signaling in response to downregulation of *a*- and *b*-series gangliosides. Western blotting indicated that the phosphorylation levels of ERK and FAK were reduced in *ST3GAL5*-siRNA-transfected HASMCs (Figure 5H), indicating that *a*- and *b*-series gangliosides contribute to the regulation of proliferation- and migration-related signaling. Therefore, attenuation of proliferation and migration in *ST3GAL5*-siRNA-transfected HASMCs was suggested to be due to the reduction of signal transduction and reduction in *cyclinD1* expression mediated by suppression of *a*- and *b*-series gangliosides.

**FIGURE 6:**
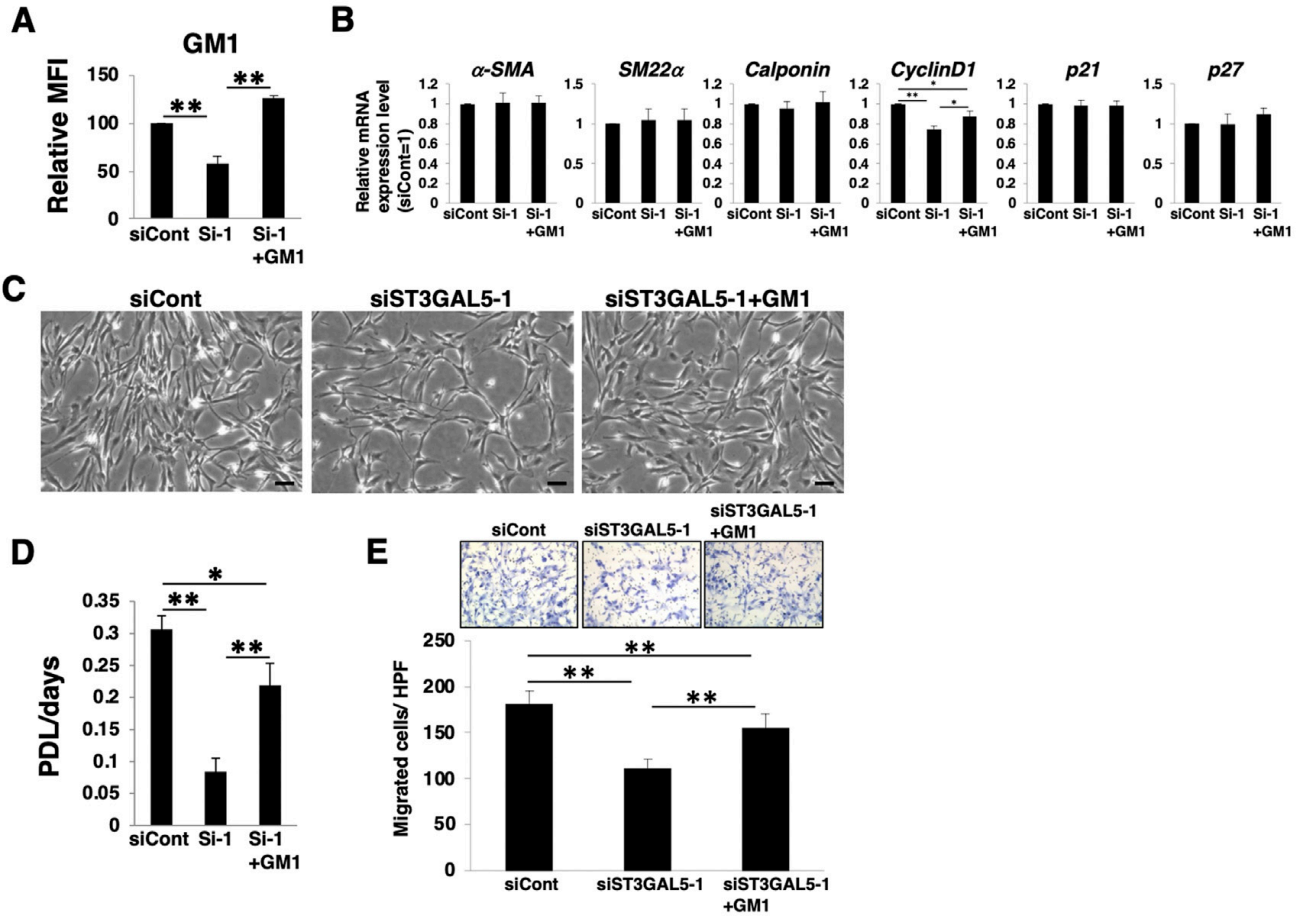
GM1 contributes to proliferation and migration of dedifferentiated human aortic smooth muscle cells (HASMCs). Differentiated HASMCs were transfected with siRNA targeting ST3GAL5 and re-cultured under normal conditions with or without 5 µM GM1 for 3 days. The cells were harvested and used in subsequent experiments. **(A)** Flow cytometry data showing the level of GM1 expression, determined as mean fluorescence intensities (MFI). Values in the cells are expressed as a percentage of corresponding values in siCont-transfected cells, which were normalized to 100%. Data are expressed as means ± standard deviation (SD) of three independent experiments. **p < 0.01 **(B)** Real-time PCR (RT-PCR) analysis of contractile or growth-related genes using cDNA derived from the cells. The results are shown as fold change relative to values in siCont-transfected cells, which are normalized to 1. Data are expressed as means ± SD of three independent experiments. *p < 0.05, **p < 0.01 **(C)** Representative phase contrast images of the cells. Scale bar: 2 µm. **(D)** Growth rate of the cells is expressed as population doubling level (PDL)/days. Data are expressed as mean ± SD of three independent experiments. *p < 0.05, **p < 0.01 **(E)** Migration capacity of the cells, expressed as the number of cells migrating across the culture insert in each high-power field (HPF). **p < 0.01, Dif, differentiated; Dedif, dedifferentiated.

Among *a*- and *b*-series gangliosides, GM1 is considered to be expressed at relatively high levels in HASMCs (Supplementary Figure S2). We then examined whether GM1 in dedifferentiated HASMCs contributes to the proliferation and migration. We added exogenously GM1 to *ST3GAL5*-siRNA-transfected HASMCs, in which GM1 was reduced. FACS analysis confirmed that the reduction of GM1 in *ST3GAL5*-siRNA-transfected HASMCs was restored by GM1 treatment (Figure 6A; Supplementary Figure S2E). Restoration of GM1 expression had no effect on smooth muscle contractile markers (*α-SMA*, *SM22α*, and *calponin*) and the cell cycle inhibitors (*p21* and *p27*), except for an increase in *cyclinD1* expression (Figure 6B). Furthermore, reduction of cell density, proliferation, and migration were restored in *ST3GAL5*-siRNA-transfected HASMCs by GM1 treatment (Figure 6C‒E). These results indicate that GM1 is involved in proliferation and migration. Taken together, it can be suggested that the relative upregulation of *a*- and *b*-series gangliosides (particularly GM1), *via* PRC2 during dedifferentiation is involved in the proliferation and migration of VSMCs.

## Discussion

In this study, we showed that the ganglioside expression changed during phenotypic switching (differentiation and dedifferentiation) of VSMCs (Figures 1, 2). Furthermore, changes in gangliosides during dedifferentiation contributed to proliferation and migration without affecting dedifferentiation. It is known that the expression patterns of sugar chains, including gangliosides, are changed during the differentiation process in stem cells, such as in embryonic stem cells (ESCs) and somatic stem cells (Ho et al., 2017; Sasaki et al., 2019a; Kimura et al., 2021). Some of these sugar chain changes are known to be functionally related to differentiation. For example, the LacdiNAc structure (GalNAcβ1,4GlcNAc) on the LIF receptor and glycoprotein 130 contributes to the regulation of LIF/STAT3 signaling for maintaining the self-renewal ability of mouse ESCs (Sasaki et al., 2011), and both *N*- and *O*-glycan processing functionally modulates the early steps of osteogenic differentiation of human mesenchymal stem cells (Wilson et al., 2018). In the future, it will be necessary to investigate other sugar chain changes (other than those in gangliosides) and their functional roles (such as involvement in dedifferentiation) in the phenotype switching of VSMCs. This will be useful for further understanding of the phenotypic switching mechanism.

We have shown that PRC2, including EZH2, is involved in the expression of gangliosides in VSMCs during differentiation or dedifferentiation. Our findings indicate that EZH2, a transcriptional repressor of H3K27 methylation, affects histone modifications in the promoter region of the *ST8SIA1* gene and controls its expression during differentiation or dedifferentiation of VSMCs. As shown in Figures 1, 2, the expression of other glycosyltransferases such as ST3GAL5 behaved similar to ST8SIA1, therefore, ST3GAL5 may be also regulated by PRC2 in VSMC. According to the Chip-atlas database, it is speculated that EZH2 may bind to the promotor lesion of ST3GAL5 and regulate ST3GAL5 expression in muscle cells. However, further experiments are required to clarify this hypothesis. Accumulating evidence suggests that PRC2 is involved in stem cell differentiation (Cao et al., 2021; Huang et al., 2021) and maintenance of the primed but not the naïve state of pluripotency (Shan et al., 2017), as well as in the regulation of sugar chain expression during the transition of embryonic stem cells to epiblast-like cells (Pecori et al., 2021). These glycosylation changes are suggested to be a result of the action of PRC2 on glycosylation-related gene expression together with other components, such as microRNAs and non-coding RNAs. In the differentiation or dedifferentiation state of VSMCs, the regulation of ganglioside expression may be modulated by PRC2-mediated ST8SIA1 expression and by the synergistic action of other components. Epigenetic factor analysis involved in ganglioside synthesis in muscle cells using the ChIP-seq dataset showed that G9a (histone H3K9 methyltransferase), HDAC1 (histone deacetylase) (Milazzo et al., 2020), EZH2, and RBBP4 (a co-repressor complex factor, such as PRC2) were highly enriched closed to the TSS (±5 kb) of genes related to ganglioside synthesis in muscle cells (Supplementary Figure S5). EZH2 and RBBP4 are well known PRC2 complex proteins (Comet et al., 2016); meanwhile, G9a and HDAC1 also bind to PRC2 as a corepressor (van der Vlag and Otte, 1999; Mozzetta et al., 2014). Therefore, although the involvement of these components in ganglioside synthesis in VSMCs is speculated, it is required to clarify their contribution in the future. Accumulating evidence suggests a critical role for HDACs in regulating vascular cell homeostasis and atherosclerosis (Chen et al., 2020; Luan et al., 2022). Sun et al. (Sun et al., 2020) reported that HDAC1 is critical for the migration and phenotypic switching of aortic VSMCs. After deacetylation of H3K27Ac, PRC2, including EZH2, catalyzes the methylation of H3K27 and induces a transcriptionally inactive heterochromatin structure (Liu, 2021). Although future studies are needed to clarify the changes in various sugar chains, including gangliosides, during VSMC differentiation or dedifferentiation, this study indicates that PRC2 and/or other epigenetic regulators, including HDAC, may be involved in the regulation of sugar chain expression.

The phenotype switching from a contractile (differentiated state) to proliferative (dedifferentiated state) phenotype is thought to be triggered by changes in local environmental cues, including an increase in the local concentration of mitogens, such as EGF, IGF, PDGF, and FGFs. Here, VSMCs dedifferentiation was induced by re-culturing differentiated cells in normal culture conditions, including 5% FBS, FGF-2, and EGF. This dedifferentiation was accompanied by an increase in the expression of *a*- and *b*-series gangliosides, which in turn contributes to proliferation and migration. In rat aortic VSMCs, overexpression of GM1 (*a*-series) and GM2 (*a*-series) promotes VSMC proliferation *via* activation of the ERK pathway (Gouni-Berthold et al., 2001). The proliferation of human VSMCs is promoted by overexpression of GD3 (*b*-series) at low concentrations (Bhunia et al., 2002). Exogenous overexpression of GD1a (*a*-series) induces increased EGF receptor (EGFR) dimerization, resulting in enhanced activation of the EGF/EGFR signaling cascade in dermal fibroblasts (Liu et al., 2004). EGF signaling promotes VSMC proliferation and migration (Zou et al., 2020). Therefore, an increase in GD1a is also suggested to promote VSMC proliferation and migration. Furthermore, GM2 contributes to the migration of cancer cells by interacting directly with integrin and modulating downstream signaling pathways, including FAK (Kundu et al., 2016). In fact, reduction of *a*- and *b*-series gangliosides in dedifferentiating VSMCs attenuates the ERK and FAK signaling pathways (Figure 5H). We also showed that exogenous addition of GM1 in *a*- and *b*-series gangliosides-reduced VSMCs restored reduced proliferation and migration (Figure 6). Currently, although it is not clear which gangliosides besides GM1 contribute to proliferation and migration, the above mechanisms mediated by increased certain *a*- and *b*-series gangliosides are considered to regulate the proliferation and migration of dedifferentiated VSMCs. Further experiments should be required for clarification of specific gangliosides involved in proliferation and migration.

**FIGURE 7:**
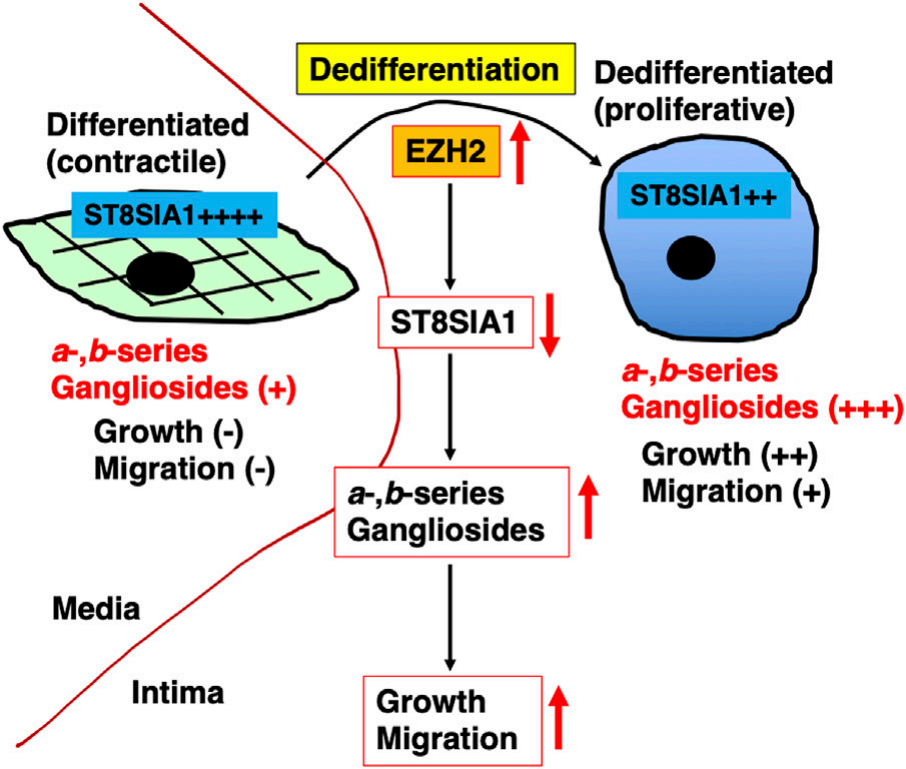
Graphic summary of this study. Phenotype switching from contractile (corresponding to medial vascular smooth muscle cells [VSMCs]) into proliferative (corresponding to intimal VSMCs) phenotype was accompanied by upregulation of *a*- and *b*-series gangliosides. These upregulations were affected by ST8SIA1, which in turn was regulated by polycomb repressor complex 2 (PRC2), including increased EZH2. The increased expression of *a*- and *b*-series gangliosides *via* PRC2 during dedifferentiation is involved in proliferation and migration.

In conclusion, we revealed the significance of gangliosides in VSMCs from the intima or media in our *in vitro* experiments in the early pathology of atherosclerosis (Figure 7). Our results indicate that PRC2 activity inhibition during phenotype switching from the contractile (medial VSMCs) to proliferative (intimal VSMCs) phenotype is useful for the inhibition of proliferation and migration mediated by inhibition of an increase in *a*- and *b*-series gangliosides. Furthermore, there are various reagents, such as HDAC inhibitors, which are also speculated to inhibit ganglioside synthesis. For instance, a specific inhibitor of ganglioside synthesis (ST3GAL5) needs to be developed in the future along with *N*-(5′-adamantane-1′-yl-methoxy)-pentyl-1-deoxynojirimycin, which is a specific inhibitor of glucosylceramide synthase that can be used to study the functional roles of endogenous gangliosides without affecting ceramide levels and used for *in vivo* analysis of ganglioside-related diseases (Aerts et al., 2003; Aerts et al., 2007). These inhibitors reduce the development of atherosclerosis in APOE*3-Leiden and low-density lipoprotein receptor^−/−^ mice (Bietrix et al., 2010). Additionally, these reagents may be useful for the atherosclerosis prevention *via* targeting gangliosides in VSMCs. In the future, *in vivo* experiments using these inhibitors could clarify the involvement of gangliosides in the early pathology of atherosclerosis. Furthermore, for the elimination of intimal VSMCs expressing gangliosides, anti-ganglioside antibodies, which are known to induce apoptosis in cancer cells (Sasaki et al., 2021), might be useful. Future *in vitro* and *in vivo* experiments are required to investigate these possibilities. Altogether, gangliosides of VSMCs are expected to be effective targets for the prevention and treatment of vascular diseases, including atherosclerosis.

## Data Availability

The raw data supporting the conclusion of this article will be made available by the authors, without undue reservation.

## Abbreviations

VSMCs, vascular smooth muscle cells; α-SMA, alpha smooth muscle actin; SM22α, smooth muscle 22 alpha; TNF, tumor necrosis factor; MMP-9, matrix metalloproteinase-9; PDGF, platelet-derived growth factor; HASMCs, human aortic smooth muscle cells; SEM, scanning electron microscope; FACS, fluorescence-activated cell sorting; ChIP, chromatin immunoprecipitation; ESCs, embryonic stem cells.

## References

[B1] Aerts J. M. Hollak C. Boot R. Groener A. (2003). Biochemistry of glycosphingolipid storage disorders: implications for therapeutic intervention. Philos. Trans. R. Soc. Lond. B Biol. Sci. 358, 905–914. 10.1098/rstb.2003.1273 12803924 PMC1693181

[B2] Aerts J. M. Ottenhoff R. Powlson A. S. Grefhorst A. Van Eijk M. Dubbelhuis P. F. (2007). Pharmacological inhibition of glucosylceramide synthase enhances insulin sensitivity. Diabetes 56, 1341–1349. 10.2337/db06-1619 17287460 PMC4298701

[B3] Alencar G. F. Owsiany K. M. Karnewar S. Sukhavasi K. Mocci G. Nguyen A. T. (2020). Stem cell pluripotency genes Klf4 and Oct4 regulate complex SMC phenotypic changes critical in late-stage atherosclerotic lesion pathogenesis. Circulation 142, 2045–2059. 10.1161/CIRCULATIONAHA.120.046672 32674599 PMC7682794

[B4] Bhunia A. K. Schwarzmann G. Chatterjee S. (2002). GD3 recruits reactive oxygen species to induce cell proliferation and apoptosis in human aortic smooth muscle cells. J. Biol. Chem. 277, 16396–16402. 10.1074/jbc.M200877200 11861654

[B5] Bietrix F. Lombardo E. Van Roomen C. P. Ottenhoff R. Vos M. Rensen P. C. (2010). Inhibition of glycosphingolipid synthesis induces a profound reduction of plasma cholesterol and inhibits atherosclerosis development in APOE*3 Leiden and low-density lipoprotein receptor-/- mice. Arterioscler. Thromb. Vasc. Biol. 30, 931–937. 10.1161/ATVBAHA.109.201673 20167657

[B6] Bobryshev Y. V. Golovanova N. K. Tran D. Samovilova N. N. Gracheva E. V. Efremov E. E. (2006). Expression of GM3 synthase in human atherosclerotic lesions. Atherosclerosis 184, 63–71. 10.1016/j.atherosclerosis.2005.04.019 15935355

[B7] Cao Y. Li L. Fan Z. (2021). The role and mechanisms of polycomb repressive complex 2 on the regulation of osteogenic and neurogenic differentiation of stem cells. Cell Prolif. 54, e13032. 10.1111/cpr.13032 33759287 PMC8088470

[B8] Cavdarli S. Groux-Degroote S. Delannoy P. (2019). Gangliosides: The double-edge sword of neuro-ectodermal derived tumors. Biomolecules 9, E311. 10.3390/biom9080311 31357634 PMC6723632

[B9] Chatterjee S. B. Dey S. Shi W. Y. Thomas K. Hutchins G. M. (1997). Accumulation of glycosphingolipids in human atherosclerotic plaque and unaffected aorta tissues. Glycobiology 7, 57–65. 10.1093/glycob/7.1.57 9061365

[B10] Chen P. Y. Qin L. Li G. Tellides G. Simons M. (2016). Fibroblast growth factor (FGF) signaling regulates transforming growth factor beta (TGFβ)-dependent smooth muscle cell phenotype modulation. Sci. Rep. 6, 33407. 10.1038/srep33407 27634335 PMC5025753

[B11] Chen X. He Y. Fu W. Sahebkar A. Tan Y. Xu S. (2020). Histone deacetylases (HDACs) and atherosclerosis: a mechanistic and pharmacological review. Front. Cell Dev. Biol. 8, 581015. 10.3389/fcell.2020.581015 33282862 PMC7688915

[B12] Comet I. Riising E. M. Leblanc B. Helin K. (2016). Maintaining cell identity: PRC2-mediated regulation of transcription and cancer. Nat. Rev. Cancer 16, 803–810. 10.1038/nrc.2016.83 27658528

[B13] Gomez D. Owens G. K. (2012). Smooth muscle cell phenotypic switching in atherosclerosis. Cardiovasc. Res. 95, 156–164. 10.1093/cvr/cvs115 22406749 PMC3388816

[B14] Gordon D. Reidy M. A. Benditt E. P. Schwartz S. M. (1990). Cell proliferation in human coronary arteries. Proc. Natl. Acad. Sci. U. S. A. 87, 4600–4604. 10.1073/pnas.87.12.4600 1972277 PMC54164

[B15] Gouni-Berthold I. Seul C. Ko Y. Hescheler J. Sachinidis A. (2001). Gangliosides GM1 and GM2 induce vascular smooth muscle cell proliferation via extracellular signal-regulated kinase 1/2 pathway. Hypertension 38, 1030–1037. 10.1161/hy1101.093104 11711493

[B16] Ho M. Y. Yu A. L. Yu J. (2017). Glycosphingolipid dynamics in human embryonic stem cell and cancer: their characterization and biomedical implications. Glycoconj. J. 34, 765–777. 10.1007/s10719-016-9715-x 27549315

[B17] Huang Y. Su T. Wang C. Dong L. Liu S. Zhu Y. (2021). Rbbp4 suppresses premature differentiation of embryonic stem cells. Stem Cell Rep. 16, 566–581. 10.1016/j.stemcr.2021.01.009 PMC7940252 33606987

[B18] Kailayangiri S. Altvater B. Lesch S. Balbach S. Gottlich C. Kuhnemundt J. (2019). EZH2 inhibition in ewing sarcoma upregulates GD2 expression for targeting with gene-modified T cells. Mol. Ther. 27, 933–946. 10.1016/j.ymthe.2019.02.014 30879952 PMC6520468

[B19] Kimura K. Koizumi T. Urasawa T. Ohta Y. Takakura D. Kawasaki N. (2021). Glycoproteomic analysis of the changes in protein N-glycosylation during neuronal differentiation in human-induced pluripotent stem cells and derived neuronal cells. Sci. Rep. 11, 11169. 10.1038/s41598-021-90102-z 34045517 PMC8160270

[B20] Kundu M. Mahata B. Banerjee A. Chakraborty S. Debnath S. Ray S. S. (2016). Ganglioside GM2 mediates migration of tumor cells by interacting with integrin and modulating the downstream signaling pathway. Biochim. Biophys. Acta 1863, 1472–1489. 10.1016/j.bbamcr.2016.04.004 27066976

[B21] Liu Y. Li R. Ladisch S. (2004). Exogenous ganglioside GD1a enhances epidermal growth factor receptor binding and dimerization. J. Biol. Chem. 279, 36481–36489. 10.1074/jbc.M402880200 15215248

[B22] Liu X. (2021). A structural perspective on gene repression by polycomb repressive complex 2. Subcell. Biochem. 96, 519–562. 10.1007/978-3-030-58971-4_17 33252743

[B23] Luan Y. Liu H. Luan Y. Yang Y. Yang J. Ren K. D. (2022). New insight in HDACs: Potential therapeutic targets for the treatment of atherosclerosis. Front. Pharmacol. 13, 863677. 10.3389/fphar.2022.863677 35529430 PMC9068932

[B24] McCabe M. T. Ott H. M. Ganji G. Korenchuk S. Thompson C. Van Aller G. S. (2012). EZH2 inhibition as a therapeutic strategy for lymphoma with EZH2-activating mutations. Nature 492, 108–112. 10.1038/nature11606 23051747

[B25] Miano J. M. Fisher E. A. Majesky M. W. (2021). Fate and state of vascular smooth muscle cells in atherosclerosis. Circulation 143, 2110–2116. 10.1161/CIRCULATIONAHA.120.049922 34029141 PMC8162373

[B26] Milazzo G. Mercatelli D. Di Muzio G. Triboli L. De Rosa P. Perini G. (2020). Histone deacetylases (HDACs): Evolution, specificity, role in transcriptional complexes, and pharmacological actionability. Genes (Basel) 11, E556. 10.3390/genes11050556 32429325 PMC7288346

[B27] Moon S. K. Kim H. M. Lee Y. C. Kim C. H. (2004). Disialoganglioside (GD3) synthase gene expression suppresses vascular smooth muscle cell responses via the inhibition of ERK1/2 phosphorylation, cell cycle progression, and matrix metalloproteinase-9 expression. J. Biol. Chem. 279, 33063–33070. 10.1074/jbc.M313462200 15175338

[B28] Mozzetta C. Pontis J. Fritsch L. Robin P. Portoso M. Proux C. (2014). The histone H3 lysine 9 methyltransferases G9a and GLP regulate polycomb repressive complex 2-mediated gene silencing. Mol. Cell 53, 277–289. 10.1016/j.molcel.2013.12.005 24389103

[B29] Oki S. Ohta T. Shioi G. Hatanaka H. Ogasawara O. Okuda Y. (2018). ChIP-atlas: a data-mining suite powered by full integration of public ChIP-seq data. EMBO Rep. 19, e46255. 10.15252/embr.201846255 30413482 PMC6280645

[B30] Park S. S. Kim W. J. Moon S. K. (2011). Suppression of vascular smooth muscle cell responses induced by TNF-alpha in GM3 synthase gene transfected cells. Int. J. Mol. Med. 27, 147–154. 10.3892/ijmm.2010.561 21072492

[B31] Pecori F. Yokota I. Hanamatsu H. Miura T. Ogura C. Ota H. (2021). A defined glycosylation regulatory network modulates total glycome dynamics during pluripotency state transition. Sci. Rep. 11, 1276. 10.1038/s41598-020-79666-4 33446700 PMC7809059

[B32] Saito M. Kitamura H. Sugiyama K. (2001). The specificity of monoclonal antibody A2B5 to c-series gangliosides. J. Neurochem. 78, 64–74. 10.1046/j.1471-4159.2001.00365.x 11432974

[B33] Sasaki N. Toyoda M. (2019). Vascular diseases and gangliosides. Int. J. Mol. Sci. 20, E6362. 10.3390/ijms20246362 31861196 PMC6941100

[B34] Sasaki A. Hata K. Suzuki S. Sawada M. Wada T. Yamaguchi K. (2003). Overexpression of plasma membrane-associated sialidase attenuates insulin signaling in transgenic mice. J. Biol. Chem. 278, 27896–27902. 10.1074/jbc.M212200200 12730204

[B35] Sasaki N. Shinomi M. Hirano K. Ui-Tei K. Nishihara S. (2011). LacdiNAc (GalNAcβ1-4GlcNAc) contributes to self-renewal of mouse embryonic stem cells by regulating leukemia inhibitory factor/STAT3 signaling. Stem Cells 29, 641–650. 10.1002/stem.615 21305673

[B36] Sasaki N. Itakura Y. Toyoda M. (2015). Ganglioside GM1 contributes to the state of insulin resistance in senescent human arterial endothelial cells. J. Biol. Chem. 290, 25475–25486. 10.1074/jbc.m115.684274 26338710 PMC4646194

[B37] Sasaki N. Itakura Y. Gomi F. Hirano K. Toyoda M. Ishiwata T. (2019a). Comparison of functional glycans between cancer stem cells and normal stem cells. Histol. Histopathol. 34, 995–1007. 10.14670/HH-18-119 31025698

[B38] Sasaki N. Itakura Y. Toyoda M. (2019b). Gangliosides contribute to vascular insulin resistance. Int. J. Mol. Sci. 20, E1819. 10.3390/ijms20081819 31013778 PMC6515378

[B39] Sasaki N. Toyoda M. Ishiwata T. (2021). Gangliosides as signaling regulators in cancer. Int. J. Mol. Sci. 22, 5076. 10.3390/ijms22105076 34064863 PMC8150402

[B40] Schnaar R. L. Sandhoff R. Tiemeyer M. Kinoshita T. (2022). “Glycosphingolipids,” in Essentials of glycobiology. Editors Varki A. Cummings R. D. Esko J. D. Stanley P. Hart G. W. Aebi M. (Cold Spring Harbor (NY): Cold Spring Harbor Laboratory Press). 35536922

[B41] Sezgin E. Levental I. Mayor S. Eggeling C. (2017). The mystery of membrane organization: Composition, regulation and roles of lipid rafts. Nat. Rev. Mol. Cell Biol. 18, 361–374. 10.1038/nrm.2017.16 28356571 PMC5500228

[B42] Shan Y. Liang Z. Xing Q. Zhang T. Wang B. Tian S. (2017). PRC2 specifies ectoderm lineages and maintains pluripotency in primed but not naive ESCs. Nat. Commun. 8, 672. 10.1038/s41467-017-00668-4 28939884 PMC5610324

[B43] Simon J. A. Lange C. A. (2008). Roles of the EZH2 histone methyltransferase in cancer epigenetics. Mutat. Res. 647, 21–29. 10.1016/j.mrfmmm.2008.07.010 18723033

[B44] Sonnino S. Chiricozzi E. Grassi S. Mauri L. Prioni S. Prinetti A. (2018). Gangliosides in membrane organization. Prog. Mol. Biol. Transl. Sci. 156, 83–120. 10.1016/bs.pmbts.2017.12.007 29747825

[B45] Sun L. Wang C. Yuan Y. Guo Z. He Y. Ma W. (2020). Downregulation of HDAC1 suppresses media degeneration by inhibiting the migration and phenotypic switch of aortic vascular smooth muscle cells in aortic dissection. J. Cell. Physiol. 235, 8747–8756. 10.1002/jcp.29718 32324261

[B46] Svennerholm L. (1980). Ganglioside designation. Adv. Exp. Med. Biol. 125, 11. 10.1007/978-1-4684-7844-0_2 7361610

[B47] van der Vlag J. Otte A. P. (1999). Transcriptional repression mediated by the human polycomb-group protein EED involves histone deacetylation. Nat. Genet. 23, 474–478. 10.1038/70602 10581039

[B48] Wang Y. Zhang Y. Gao X. Qian J. Yang J. Sun W. (2021). Resistin-like molecule beta augments phenotypic modulation of human aortic smooth muscle cell triggered by high glucose. Endocr. J. 68, 461–468. 10.1507/endocrj.EJ20-0343 33441498

[B49] Wilson K. M. Jagger A. M. Walker M. Seinkmane E. Fox J. M. Kröger R. (2018). Glycans modify mesenchymal stem cell differentiation to impact on the function of resulting osteoblasts. J. Cell Sci. 131, jcs209452. 10.1242/jcs.209452 29361539 PMC5868951

[B50] Zhang L. Chen Q. An W. Yang F. Maguire E. M. Chen D. (2017). Novel pathological role of hnRNPA1 (heterogeneous nuclear ribonucleoprotein A1) in vascular smooth muscle cell function and neointima hyperplasia. Arterioscler. Thromb. Vasc. Biol. 37, 2182–2194. 10.1161/ATVBAHA.117.310020 28912364 PMC5660626

[B51] Zhang F. Guo X. Xia Y. Mao L. (2021a). An update on the phenotypic switching of vascular smooth muscle cells in the pathogenesis of atherosclerosis. Cell. Mol. Life Sci. 79, 6. 10.1007/s00018-021-04079-z 34936041 PMC11072026

[B52] Zhang J. Guo J. R. Wu X. L. Wang X. Zhu Z. M. Wang Y. (2021b). TWIST1 induces phenotypic switching of vascular smooth muscle cells by downregulating p68 and microRNA-143/145. FEBS Open Bio 11, 932–943. 10.1002/2211-5463.13092 PMC7931233 33470057

[B53] Zhuo D. Guan F. (2019). Ganglioside GM1 promotes contact inhibition of growth by regulating the localization of epidermal growth factor receptor from glycosphingolipid-enriched microdomain to caveolae. Cell Prolif. 52, e12639. 10.1111/cpr.12639 31127673 PMC6668969

[B54] Zou Z. G. Rios F. J. Neves K. B. Alves-Lopes R. Ling J. Baillie G. S. (2020). Epidermal growth factor signaling through transient receptor potential melastatin 7 cation channel regulates vascular smooth muscle cell function. Clin. Sci. (Lond.) 134, 2019–2035. 10.1042/CS20200827 32706027 PMC8299307

