# Supplementary figures and images for: PRC2-dependent regulation of ganglioside expression during dedifferentiation contributes to the proliferation and migration of vascular smooth muscle cells
Source: Front Cell Dev Biol. 2022 Oct 13;10:1003349. doi: 10.3389/fcell.2022.1003349 (PMC9606594; doi:10.3389/fcell.2022.1003349)

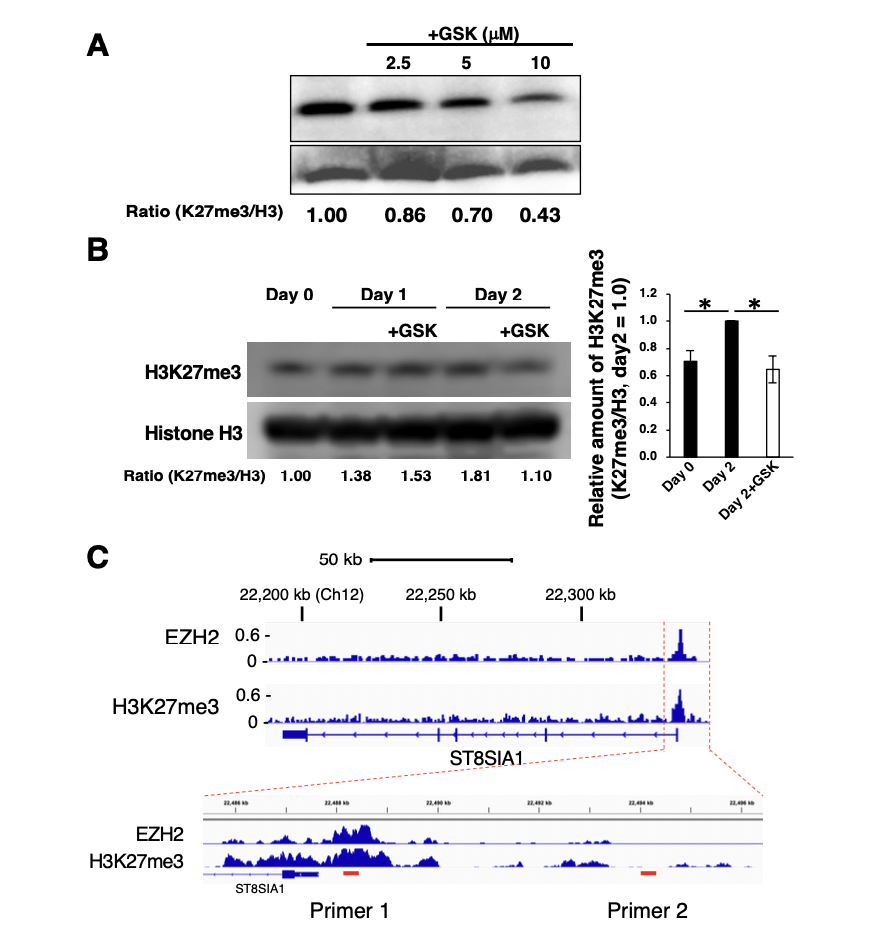

Supplement: Supplementary file 1 [file Image1.tiff]
