# Supplementary material for: PRC2-dependent regulation of ganglioside expression during dedifferentiation contributes to the proliferation and migration of vascular smooth muscle cells
Source: Front Cell Dev Biol. 2022 Oct 13;10:1003349. doi: 10.3389/fcell.2022.1003349 (PMC9606594; doi:10.3389/fcell.2022.1003349)
Supplement: Supplementary file 2 [file DataSheet1.docx]

Supplementary Material

**Supplementary Figure S1.** Main synthetic pathway of *a*- and *b*-series gangliosides. Glycosyltransferases contributing to each synthetic pathway are also shown.

**Supplementary Figure S2.** Histograms supporting the FACS analysis of gangliosides are for the results shown in (**A**) Figure 1G, (**B**) Figure 2F, (**C**) Figure 4F, (**D**) Figure 5B, and (**E**) Figure 6A. Representative results are shown. Positive histograms are shown using black lines, and negative controls are depicted as the graphs filled with light gray color.

**Supplementary Figure S3.** (**A**) Western blot analysis of H3K27me3 expression in differentiated human aortic smooth muscle cells (HASMCs) re-cultured under normal conditions with or without 2.5, 5, or 10 μM GSK126 for 3 days. Ratio of densitometric values is shown below the respective protein band images (non-treated = 1.00). (**B**) Western blot analysis of H3K27me3 expression in differentiated HASMCs re-cultured under normal conditions with or without 10 μM GSK126 for 0–2 days. The histograms show mean densitometric readings ± SD for H3K27me3 normalized to the loading controls (Histone H3). Results are expressed as means ± SD of three independent experiments. **P* < 0.05. (**C**) Schematic representation of the proximal region of the human *ST8SIA1* gene (human genome: hg19 ch12). Histogram of EZH2 and H3K27me3 expression in rhabdomyosarcoma was obtained from the chromatin immunoprecipitation (ChIP)-atlas (http://chip-atlas.org/). Two sets of PCR primers designated as Primer 1 (predicted EZH2-regulated region) and Primer 2 (EZH2-independent region) on the *ST8SIA1* promoter region were used for ChIP analysis.

**Supplementary Figure S4.** Immunoblotting for the contractile markers in the cells. The blot images were cropped to highlight the α-SMA, SM22α, and β-ACTIN bands. Ratio of the densitometric values is shown below the respective protein band images (Dif. = 1.00). Dif.: differentiation, Dedif.: dedifferentiation.

**Supplementary Figure S5.** Analyzed chromatin immunoprecipitation-seq datasets were obtained from untreated muscular cell lines precipitated using anti-epigenetic factors: anti-G9a (EHMT2) Ab (SRX4561210, RH-41), anti-HDAC1 Ab (SRX4313223, RH-4), anti-EZH2 Ab (SRX1998402, Rhabdomyosarcoma), anti-RBBP4 Ab (SRX7115500, RH-4), anti-HDAC2 Ab (SRX4313202, RH-4), anti-YY1 Ab (SRX4313226, RH-4), anti-EP300 Ab (SRX1878906, Rh-4), anti-BRD4 Ab (SRX1878887, Rh-4), and anti-HDAC3 Ab (SRX5930140, RH-4). Percentage of ganglioside synthesis-genes occupied by epigenetic factors was determined within a range of ± 5 kb, with a threshold for statistical significance calculated by peak-caller MACS2 set as 100 (1 < 1E−05).

**Supplementary Figure S6.** Western blots. Protein bands and molecular weight markers are presented for (**A**) Figure 2H, (**B**) Figure 3C, (**C**) Figure 3D, and (**D**) Figure 4J.

**Supplementary Table S1.** List of primers for real-time PCR

| **Gene** | **Forward primer** | **Reverse primer** |
| --- | --- | --- |
| *ST3GAL5* | AGGAATGTCGTCCCAAGTTTG | GGAGTAAGTCCACGCTATACCT |
| *B4GALNT1* | ACAGCAGACACAGTCCGGTTCT | GCGGGTGTCTTATGCGGATA |
| *ST8SIA1* | TACTCTCTCTTCCCACAGG | GACAAAGGAGGGAGATTGC |
| *B3GALT4* | GAAGGAGGCCAGGTTTTGC | CCCGGCCCAAGTACAGAAG |
| *ST3GAL2* | TGGACGGGCACAACTTCA | TGCCAACATCCTGCTCAAAG |
| *ST8SIA5* | CCATGGTGAAGCAGTCAGAGCTG | GGAGTGTTCTTCTGGGTGGTGAA |
| *NEU3* | AATGTGAAGTGGCAGAGGTGA | TCACAGAGCTGTCGACTCAGG |
| *EZH2* | TGGGCAATTTAGAAAAAGAACATGC | GCAGCTGGTGAGAAGGCAATA |
| *α-SMA* | CACCATCGGAAATGAACGTTT | GACTCCATCCCGATGAAGGA |
| *SM22α* | GGCGTGATTCTGAGCAAGCT | CACCTTCACCGGCTTGGA |
| *Calponin* | AGAACAAGCTGGCCCAGAAG | CCCCTCGATCCACTCTCTCA |
| *Cyclin D1* | GCGAGGAACAGAAGTGC | GAGTTGTCGGTGTAGATGC |
| *p21* | TGGAGACTCTCAGGGTCGAAA | GCGTTTGGAGTGGTAGAAATCTG |
| *p27* | AGACTGATCCGTCGGACAGC | CACAGAACCGGCATTTGGG |
| *β-ACTIN* | GGTCATCACCATTGGCAATGAG | TACAGGTCTTTGCGGATGTCC |

**Supplementary Table S2.** List of primers for chromatin immunoprecipitation analysis

|  |  | hST8SIA1_ChIP-1 |
| --- | --- | --- |
| F | 5' | GGCGGAGATTTATTCCTTCC 3ʹ |
| R | 5' | CACATCTGGATGCAGCACTT 3ʹ |
|  |  | hST8SIA1_ChIP-2 |
| F | 5' | TGATGTAAGCAGAGGCATGG 3ʹ |
| R | 5' | TTGTCCTGTGGTTCAGCTTG 3ʹ |
